# Biocompatible Platinum Nanoclusters Prepared Using Bitter Gourd Polysaccharide for Colorimetric Detection of Ascorbic Acid

**DOI:** 10.3390/biom11050647

**Published:** 2021-04-28

**Authors:** Kai Liu, Yu Zhao, Lu Zhang, Mengmeng He, Weifeng Lin, Haotian Sun, Zhiwei Liu, Jie Hu, Longgang Wang

**Affiliations:** 1 State Key Laboratory of Metastable Materials Science and Technology, Key Laboratory of Applied Chemistry, Hebei Key Laboratory of Heavy Metal Deep-Remediation in Water and Resource Reuse, Yanshan University, Qinhuangdao 066004, China; kailiu241819@163.com (K.L.); zhaoy9402@163.com (Y.Z.); ZL1434901904@163.com (L.Z.); hmm981211@163.com (M.H.); zwliu@ysu.edu.cn (Z.L.); hujie@ysu.edu.cn (J.H.); 2 Department of Molecular Chemistry and Materials Science, Weizmann Institute of Science, Rehovot 76100, Israel; lin.weifeng@weizmann.ac.il; 3 Ocean NanoTech, LLC, San Diego, CA 92126, USA; hsun9@buffalo.edu

**Keywords:** green synthesis, colorimetric, bitter gourd polysaccharide, ascorbic acid, detection

## Abstract

Ascorbic acid is an organic compound with antioxidant properties that can protect the human body from the threat of free radicals. Therefore, it is important to detect the existence and measure the concentration of ascorbic acid to regulate its content in the human body. In this work, we prepared bitter gourd polysaccharide (BGP)-stabilized platinum nanoclusters (Pt-BGP NCs) by reacting BGP with K_2_PtCl_4_. Pt-BGP NCs and catalyzed the decomposition of H_2_O_2_ to generate •OH radicals, which could oxidize TMB to form oxidized TMB (oxTMB), indicating their peroxidase-like properties. The kinetics followed the Michaelis–Menten equation. Furthermore, the colorimetric detection of ascorbic acid using Pt-BGP NCs showed high selectivity and a low detection limit of 0.191 μM. The accuracy of real sample detection using Pt-BGP NCs was as high as 98.9%. More importantly, Pt-BGP NCs had high cell biocompatibility and extremely low hemolysis rate due to the component of BGP. In summary, the prepared Pt-BGP NCs with reductive activity and good biocompatibility have good application prospects in colorimetric detection of ascorbic acid.

## 1. Introduction

Ascorbic acid acts as a biological cofactor that is abundantly present in fruits and vegetables. It plays an important role in many biochemical processes; thus, it is essential for human health. Ascorbic acid has antioxidant properties, and a lack or imbalance of ascorbic acid is related to the symptoms of many diseases, such as cardiovascular disease or cancer [1]. The concentration of ascorbic acid in the central nervous system is at millimolar level, while its concentration is very low in other body fluids. Ascorbic acid has been increasingly used in industries due to its strong antioxidant capacity. Therefore, it is necessary to develop a simple, rapid and highly sensitive ascorbic acid detection method for application in food, pharmaceutical and cosmetic industries.

There are a variety of methods for the detection of ascorbic acid, including electrochemical methods, chromatography, fluorescence and chemiluminescence [2]. Colorimetric detection is a more ideal method due to its specification, simplicity and practicality. The application of colorimetric detection depends on natural enzymes such as peroxidase. Recently, many nanomaterials, such as ZnO nanoparticles [3], conjugated microporous polymer (CMP-LS9) [4] and porous carbon nanozymes [5], have been used in colorimetric detection base on their enzyme-like catalytic ability. Liu et al. [6] used 3,4:9,10-perylene tetracarboxylic acid (PTCA) modified litchi-like zinc ferrite (ZnFe_2_O_4_) to prepare nanocomposite material, which was used for the colorimetric detection of ascorbic acid. The final linear range of ascorbic acid detection is 1–10 μM, and the detection limit is 0.834 μM. The detection limit is high due to the low activity of the artificial enzymes employed in their study, and the detection range of this method is also not wide enough. It is obviously feasible to reduce the size of the artificial enzymes and improve their specific surface area to enhance detection performance. At the same time, most mimetic enzymes have poor stability in water; improving their stability in water could also improve the catalytic activity.

Bitter gourd is one kind of common vegetable, and it has been used as a drug for the treatment of diabetes in Asia [7]. Bitter gourd polysaccharide is considered to be the main ingredient for medicinal purposes. Panda et al. [8] prove bitter gourd polysaccharide (BGP) has the effect of enhancing immunity and anti-oxidation. The hydrolysate of bitter gourd polysaccharide mainly contains glucose (35.3%), galactose (28.2%), arabinose (10.1%), mannose (9.6%), rhamnose (7.2%), xylose (5.3%) and fucoid sugar (2.1%). The reducing groups of BGP, such as aldehyde group, can gradually reduce platinum ion to generate Pt nanoparticles, which will be stabilized by soluble BGP [9]. Bitter gourd polysaccharide has high stability and water solubility, which is an ideal biological template to stabilize metal nanoparticles.

In this work, we extracted bitter gourd polysaccharide from bitter gourd by hydrothermal extraction. Bitter gourd polysaccharide stabilized platinum nanoclusters (Pt-BGP NCs) were prepared, where bitter gourd polysaccharide was used as a reducing agent and stabilizer. Pt-BGP NCs were successfully prepared by the green synthetic method, which had the advantages of simple preparation using nontoxic and environment-friendly agents [10]. The Pt-BGP NCs had ultra-small size as platinum nanoclusters (Pt NCs), good stability, high catalytic activity and good biocompatibility, all of which were due to the reducing ability, water solubility and good biocompatibility of bitter gourd polysaccharide. Pt-BGP NCs effectively catalyzed the decomposition of H_2_O_2_ to produce •OH radicals, which oxidized TMB to form blue oxTMB. Ascorbic acid selectively reduced the blue oxTMB to colorless TMB. Therefore, a simple and sensitive method was established to detect ascorbic acid concentration. The measurement of the concentration of ascorbic acid in tablets confirmed that Pt-BGP NCs had great potential in real samples for biomedical related tests.

## 2. Materials and Methods

### 2.1. Materials

Bitter gourd polysaccharide was extracted according to a method reported by Yan [11]. Potassium tetrachloroplatinate (K_2_PtCl_4_), 3,3′,5,5′-tetramethylbenzidine (TMB), acetic acid, sodium acetate, iron(III) chloride hexahydrate, sodium chloride, sodium hydroxide, chlorine potassium, potassium dihydrogen phosphate, disodium hydrogen phosphate, terephthalic acid (TA), H_2_O_2_, thiazole blue (MTT), and ascorbic acid were purchased from Aladdin (Shanghai, China). HeLa cells are a human cervical cancer cell line. Dialysis bags (MWCO = 14,000) were purchased from Spectrum Laboratories Inc (Piscataway, NJ, USA).

### 2.2. Synthesis and Characterization of Pt-BGP NCs

BGP and K_2_PtCl_4_ were dissolved in deionized water at concentrations of 1 mg/mL and 2 mM, respectively. Then, the two solutions were mixed, where the molar ratio of BGP to K_2_PtCl_4_ was 1:25 and the concentration of Pt was 0.66 mM. The mixed solution was placed in a Thermal Shaker at 80 °C for 12 h. The solution was further dialyzed against deionized water for 24 h to obtain Pt-BGP NCs. Then, the prepared Pt-BGP NCs solution was stored in a refrigerator at 4 °C.

The reducing power of BGP was characterized according to a previous method reported by Yan et al. [12]. The molecular weight of the BGP was measured by gel permeation chromatography (GPC, Kyoto, Shimadzu, Japan), the gel column was filled with dextran and eluted with ultrapure water, and the elution curve and the relative molecular weight of the polysaccharide were measured. A Fourier infrared spectrometer (TU-1901/1900, Karlsruhe, Bruker, Germany) was used to measure the infrared spectra of the BGP and Pt-BGP NCs samples. A transmission electron microscope (TEM, JEM-1230EX, Tokyo, Hitachi, Japan) was used to observe the morphology of Pt NCs and measure the particle size. The hydrodynamic sizes of Pt-BGP NCs in different pH buffer solutions were measured by dynamic light scattering (DLS, Worcestershire, Malvern, UK).

The catalytic mechanism was tested with the following steps. First, we set up 4 groups of experiments: TA, TA + Pt-BGP NCs, TA + H_2_O_2_ and TA + H_2_O_2_ + Pt-BGP NCs. As for TA + H_2_O_2_ + Pt-BGP NCs, 900 μL of TA (C_TA_ = 0.5 mM) was added into a 2 mL PE tube, and then 200 μL of HAc-NaAc buffer solution (pH = 4) was added into the above PE tube. Finally, 300 μL of Pt-BGP NCs (C_Pt_ = 0.66 mM) and 100 μL of H_2_O_2_ (300 mM) solution was taken into the tube. The mixed solution reacted at 25 °C for 12 h. The fluorescence spectra of final solution were measured with a fluorescence spectrometer (Tokyo, Hitachi, Japan). The other control groups were carried out through the same procedure.

### 2.3. Determination of Ascorbic Acid Concentration

To establish the standard curve, the test system consisted of 100 μL of Pt-BGP NCs (C_Pt_ = 0.66 mM), 900 μL of TMB (pH = 4, 0.6 mM), and 100 μL of H_2_O_2_ (0.3 M), and they were mixed at 30 °C for 5 min. Then, 100 μL of ascorbic acid at different concentrations was added, the mixtures were further incubated at 40 °C for 5 min, and the absorbance was measured at 652 nm. The time required for this detection was 10 min. The absorbance of interfering agents (0.1 mM), such as Na^+^, Fe^3+^, K^+^, glucose, alanine, histidine, lactose and BSA, was measured using the same method. The pills were dissolved in water and measured using established method.

### 2.4. Biocompatibility of Pt-BGP NCs

The MTT experiment was processed according to the experimental process of Cui et al. [13]. Human red blood cells (RBC) from healthy volunteers were isolated. Then, a 2% w/v phosphate solution saline (PBS) containing human red blood cells was prepared. A total of 500 μL of sample with RBC was mixed at a volume ratio of 1:1 and incubated at 37 °C for 4 h. At the same time, PBS and deionized water were set as negative control and positive control, respectively. Each sample solution was transferred to a 96-well plate and measured with a microplate reader at 575 nm.

## 3. Results

### 3.1. Characterization of GBP

**Figure 1:**
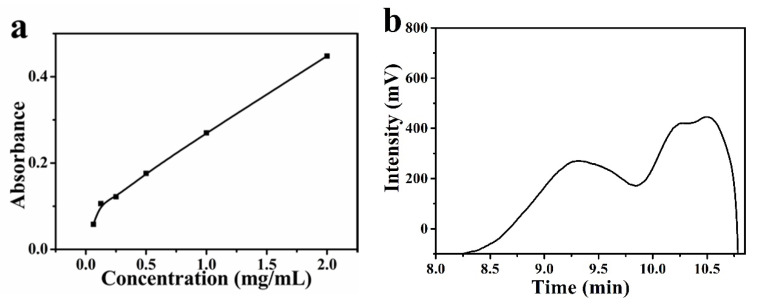
(**a**) The reducing power of BGP and (**b**) GPC elution curve of BGP.

The reducing group could reduce the Fe^3+^ to Fe^2+^ in potassium ferricyanide; potassium ferrocyanide and ferric chloride would form Fe_4_[Fe(CN)_6_]_3_ which has the maximum absorption peak at 700 nm. Absorbance at 700 nm is positively correlated with the reducing power of the material. As shown in Figure 1a, the absorbance of the mixed solution at 700 nm increased with the increase of BGP concentration. When the concentration of BGP reached 2 mg/mL, its absorbance reached 0.448. Chen et al. [14] prepare phosphorylated pumpkin polysaccharide and test its antioxidant capacity and reducing capacity. In their study, the absorbance of Fe_4_[Fe(CN)_6_]_3_ is only 0.1 at 700 nm at concentration of 2 mg/mL. Figure 1b showed the curve of BGP retention time. The weight average molecular weight of BGP was 27,957 Da.

Protein in BGP has an impact on subsequent experiments, and protein content of BGP needs to be tested. Protein content was measured by the Coomassie brilliant blue method. Based on this method, Gu et al. [15] find that the protein content of the extracted ginseng polysaccharide is about 1.46 %. As shown in Figure S1, the regression equation of the standard curve obtained after linear fitting was y = 6.1417x + 0.17694 (R^2^ = 0.986). The purity of BGP in the polysaccharide was 99.99%.

### 3.2. Characterization of Pt -BGP NCs

**Figure 2:**
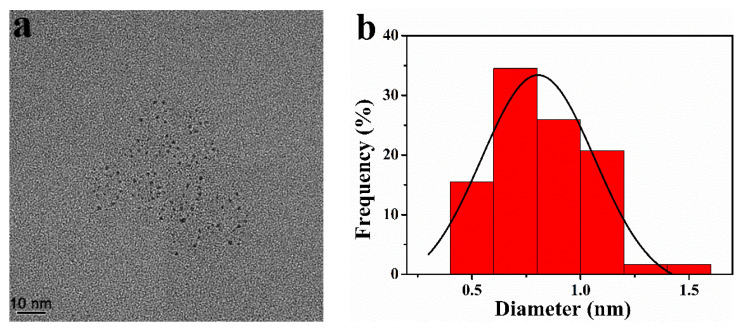
(**a**) HRTEM image and (**b**) size distribution of Pt NCs inside of Pt-BGP NCs.

The size of Pt NCs inside Pt-BGP NCs was measured by HRTEM. Figure 2a revealed that the Pt NCs had good dispersibility and were spherical in shape. Statistics showed that the size of Pt-BGP NCs was 0.83 ± 0.22 nm (Figure 2b).

**Figure 3:**
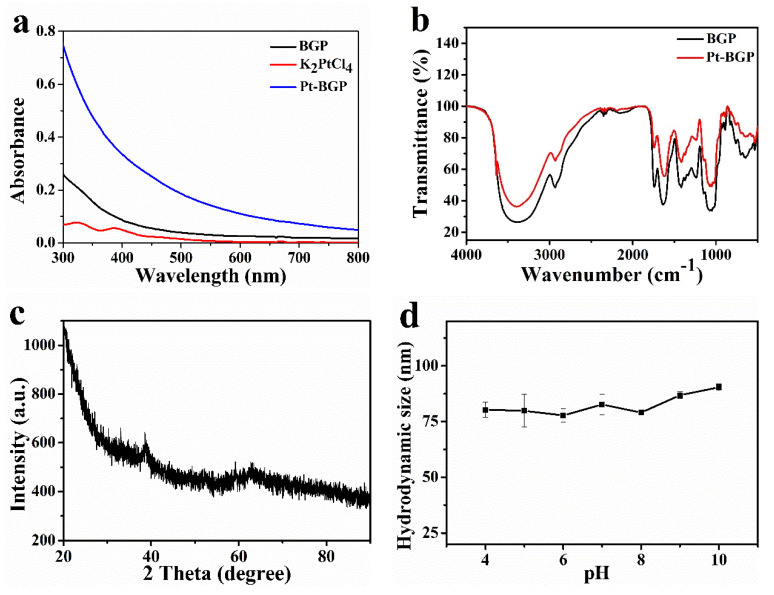
(**a**) UV-Vis spectra of BGP, K_2_PtCl_4_ and Pt-BGP NCs; (**b**) FTIR spectra of BGP and Pt-BGP NCs; (**c**) XRD diffraction pattern of Pt-BGP NCs; (**d**) hydrodynamic size of Pt-BGP NCs in different pH solutions.

UV-Vis spectra were utilized to judge the formation of Pt-BGP NCs. Figure 3a showed that the two small absorption peaks of K_2_PtCl_4_ were at 325 nm and 388 nm, respectively. After incubating with K_2_PtCl_4_ and BGP for 12 h, both peaks disappeared, proving that there was no K_2_PtCl_4_ in the mixed solution. Moreover, the wide and long absorption band of Pt-BGP NCs was obviously different from BGP. The Pt nanoparticles prepared by Dobrucka et al. [16] with plantain extract also exhibit a similar full-wavelength UV-Vis spectra, which indicated that Pt NCs had been stabilized by BGP.

In order to explore the changes of functional groups after the reduction and formation of Pt NCs by BGP, infrared spectroscopy analysis was performed. As shown in Figure 3b, the broad absorption peak at 3410 cm^−1^ was the stretching vibration of the O-H bond, which indicated the presence of intramolecular and intermolecular hydrogen bonds in the polysaccharide molecule. -CH_2_ was observed under the stretching vibration of 2930 cm^−1^. The weak absorption peak at 1735 cm^−1^ corresponded to the characteristic peak of carboxylic acid in glucuronic acid. C=O tensile vibration was observed at 1635 cm^−1^, which was a characteristic absorption peak of carbohydrates. The absorption peak at 1420 cm^−1^ was led by the C-H asymmetric stretching vibration, while the absorption peak near 1050 cm^−1^ was caused by the C-O stretching vibration on the carboxyl group, sugar ring and glycosidic bond. A characteristic absorption peak at 890 cm^−1^ was observed, which indicated that there was a β-glycosidic bond. There was no obvious change in the infrared peak pattern between Pt-BGP NCs and BGP. This was similar to the infrared spectra of BGP extracted by Huang et al. [17].

The crystal form and spatial structure of Pt-BGP NCs were characterized by XRD. As shown in Figure 3c, there was a weak reflection at the 38.85° (111) crystal plane (JCPDS: 04-0802). This might be due to the small size of the generated nanoclusters. DLS was used to characterize the hydrodynamic size of Pt-BGP NCs in different pH solutions. As shown in Figure 3d, the hydrodynamic size of Pt-BGP NCs maintained about 80 nm in different pH solutions.

### 3.3. Peroxidase-Like Activity and Catalytic Mechanism

**Figure 4:**
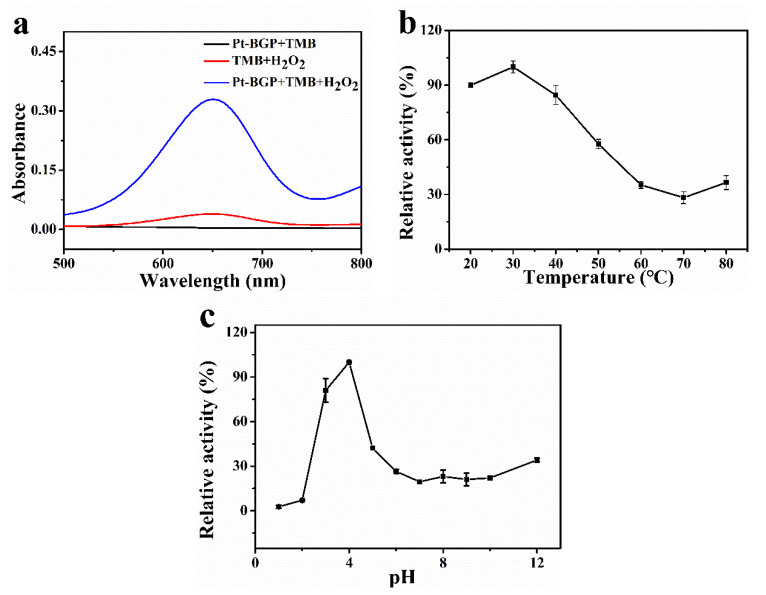
(**a**) UV-Vis spectra of different sample solutions; effect of (**b**) temperature and (**c**) pH on the catalytic activity of Pt-BGP NCs.

To study the catalytic activity of Pt-BGP NCs, three groups, Pt-BGP + TMB, TMB + H_2_O_2_ and Pt-BGP + TMB + H_2_O_2_, were carried out. As shown in Figure 4a, Pt-BGP + TMB and TMB + H_2_O_2_ had very low absorbance at 652 nm. While Pt-BGP + TMB + H_2_O_2_ had an obvious peak at 652 nm. This indicated that, when Pt-BGP NCs participated in the reaction between TMB + H_2_O_2_, the reaction significantly improved, which demonstrated that Pt-BGP NCs had obvious peroxidase-like activity. Pt-BGP NCs might accelerate the decomposition of H_2_O_2_ to generate oxygen species (ROS) with enhanced oxidation ability, and ROS quickly oxidized TMB. Liu et al. [18] used a simple hydrothermal method to synthesize highly dispersed ultrafine IrO_2_ nanoparticles on reduced graphene oxide (rGO) nanosheets, and explored their peroxidase-like activity.

As we know, catalysts have the best catalytic effect when they are under optimal conditions. Therefore, the influence of temperature and pH on Pt-BGP NCs was explored. Figure 4b showed that Pt-BGP NCs had maximum activity at 30 °C. When the temperature was less than 50 °C, all had more than 50% activity. Figure 4c illustrates that Pt-BGP NCs had maximum catalytic activity at pH = 4, while they had very low activity when pH was higher than pH = 6.

**Figure 5:**
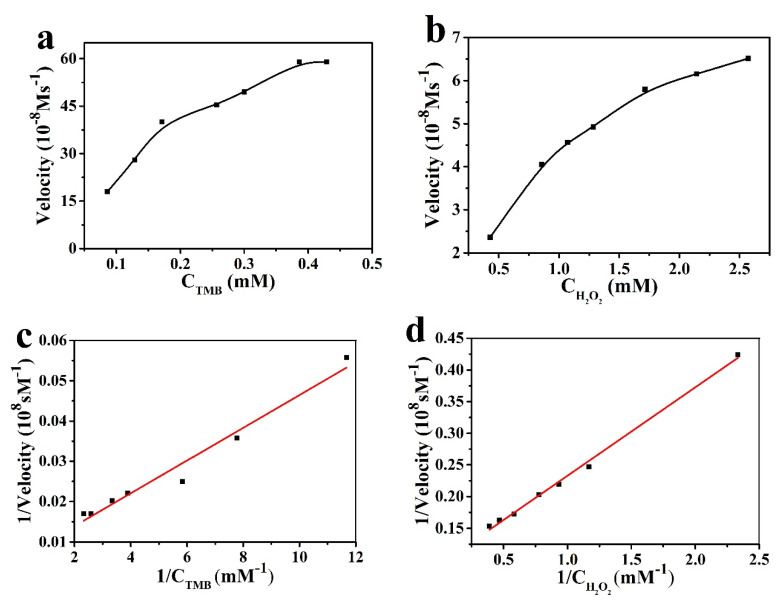
The catalytic kinetics of Pt-BGP NCs for (**a**) TMB and (**b**) H_2_O_2_; (**c**,**d**) was the reciprocal of (**a**,**b**).

In order to compare the catalytic performance of the mimic enzyme and the natural enzyme, the catalytic performance of Pt-BGP NCs was tested. Figure 5a,b shows the Michaelis–Menten curves, with TMB and H_2_O_2_ as substrates, respectively. The V_max_ of Pt-BGP NCS and HRP towards H_2_O_2_ was 10.73 × 10^−8^ Ms^−1^and 8.71 × 10^−8^ Ms^−1^, respectively. Hence, Pt-BGP NCs had higher catalytic performance towards H_2_O_2_ than HRP. Huang et al. [19] pyrolyze the metal–organic framework made of cerium (III) and trimesic acid to prepare CeO_2_/C nanowires (CeO_2_/C NWs). The K_m_ and V_max_ of CeO_2_/C NWs to TMB were 0.12 mM and 2.08 × 10^−8^ Ms^−1^, respectively. The K_m_ and V_max_ of Pt-BGP NCs towards TMB were 0.70 mM and 175.44 × 10^−8^ Ms^−1^, respectively. Compared with CeO_2_/C NWs, Pt-BGP NCs had a higher K_m_ and V_max_ towards TMB. Table 1 shows the K_m_ and V_max_ of Pt-BGP NCs, HRP and other mimic enzymes.

**Figure 6:**
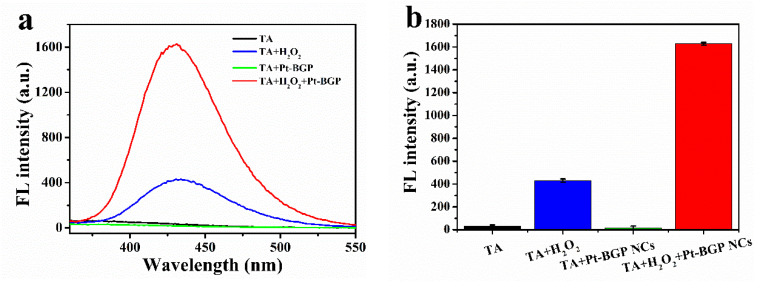
(**a**) Fluorescence spectra of TA, TA + H_2_O_2_, TA + Pt-BGP NCs and TA + H_2_O_2_ + Pt-BGP NCs after incubation for 12 h; (**b**) histograms of fluorescence intensity at 435 nm.

H_2_O_2_ in the presence of HRP would generate •OH radicals, which are finally decomposed into water and oxygen. Similarly, the peroxidase activity of Pt-BGP NCs might depend on the ability of Pt-BGP NCs to generate •OH radicals in an H_2_O_2_ solution. To confirm this hypothesis, TA, as a fluorescent probe, was used to test the generation of •OH radicals. The fluorescence spectrum was collected at the excitation wavelength of 315 nm. The intensity of the fluorescence spectrum of 2-hydroxyterephthalic acid was directly proportional to the amount of •OH radicals. Therefore, we conducted four sets of experiments, as shown in Figure 6a,b: TA, TA + H_2_O_2_, TA + Pt-BGP NCs and TA + H_2_O_2_ + Pt-BGP NCs. It can be seen from Figure 6 that the fluorescence intensity of TA, TA + H_2_O_2_, TA + Pt-BGP NCs and TA + H_2_O_2_ + Pt-BGP NCs was 31.9, 429.9, 16.7, 1629.0, respectively.

### 3.4. Determination of Ascorbic Acid Concentration

**Figure 7:**
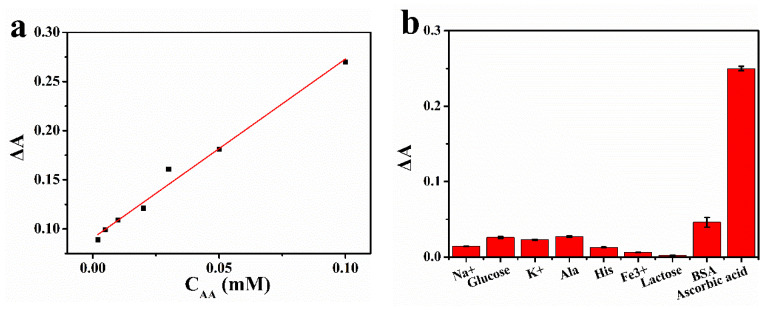
(**a**) The standard curve for the detection of ascorbic acid concentration using Pt-BGP NCs; (**b**) selective detection of ascorbic acid.

**Figure 8:**
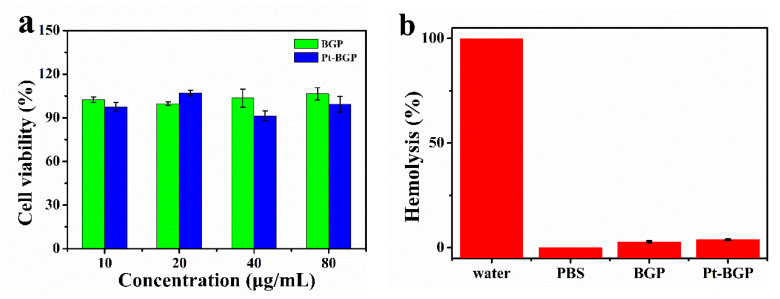
(**a**) Cell viability of HeLa cell after treatment with BGP and Pt-BGP NCs; (**b**) hemolysis rate and corresponding photos of red blood cells incubated with BGP and Pt-BGP NCs (100 μg/mL) at 37 °C for 4 h, respectively.

Ascorbic acid undergoes a redox reaction with oxidized TMB to produce colorless TMB. Therefore, the concentration of ascorbic acid was positively correlated with the change of absorbance of oxTMB. Figure 7a showed that the relationship between the absorbance of mixed solution at 652 nm and the concentration of ascorbic acid (2–100 μM) was linear, and this upper limit was a relatively high concentration in the existing literature [24]. The linear regression equation was ΔA = 1.823C_AA_ + 0.0906 (R^2^ = 0.993), and the detection limit was 0.191 μM. The detection limit of this catalyst was slightly lower than that of other catalysts (Table 2). As shown in Figure 8b, the selectivity of this method was tested with Na^+^, Fe^3+^, K^+^, alanine, histidine, glucose, lactose and BSA.

In order to detect ascorbic acid in real samples by the established method of using Pt-BGP NCs, medical vitamin C (*Vc*) tablets were purchased from pharmacies and dissolved. Ascorbic acid concentration in the samples was measured.

### 3.5. Biocompatibility

BGP has been proven to have many biological activities, such as antioxidant activity and biocompatibility [28]. Here, the cytotoxicity of Pt-BGP NCs and BGP was explored through an MTT assay. As shown in Figure 8a, when the concentration of Pt-BGP NCs and BGP was as high as 80 μg/mL, the cell viability of HeLa cells was still about 100%. This indicated that BGP and Pt-BGP NCs at this concentration had no obvious cytotoxicity.

The study of hemolysis analysis of red blood cells was performed to evaluate biocompatibility with red blood cells. Figure 8b shows that the solution turned red when red blood cells were dispersed in water, and the red substance was hemoglobin. When the sample concentration was 100 μg/mL, the hemolysis rates of BGP and Pt-BGP NCs were 2.89% and 3.79%.

## 4. Discussion

Compared with other polysaccharides, BGP had a better reduction ability due to hydroxyl and aldehyde groups, which indicated that BGP could be used in the reduction preparation of nanomaterials as a reducing agent. GPC was used to determine the molecular weight of BGP. The weight average molecular weight was 27,957 Da. The protein content of BGP was 0.01%; the protein in BGP will not affect subsequent experiments.

The size of Pt-BGP NCs was 0.83 ± 0.22 nm, which is smaller than other Pt NCs reported in the literature. Parisa et al. [29] use Gram-negative ADR19, FZC6 and B-11177 bacteria to synthesize Pt nanoparticles, with an average size of 3.95, 2.49 and 3.84 nm, respectively. Kora et al. [20] use gumolibanum (Boswellia serrata) to stabilize platinum nanoparticles, whose size is around 4.4 nm. Compared with these materials, the size of Pt NCs inside of Pt-BGP NCs was obviously smaller, because BGP had a strong reducing power which accelerated the formation of Pt NCs. Pt NCs inside of Pt-BGP NCs had larger specific surface area, which might improve their catalytic activity. Pt-BGP NCs had good stability due to the existence of BGP as a stabilizer by DLS.

Pt-BGP NCs had peroxidase-like activity by studying the catalytic activity of Pt-BGP NCs. Pt-BGP NCs had maximum catalytic activity at pH = 4 and 30 °C. The optimal temperature for the multilayer CeO_2_ coated Ag_2_S microspheres prepared by Lian et al. [30] is 55 °C, which is higher than Pt-BGP NCs. Pt-BGP NCs showed maximum reactive activity at a mild temperature. Compared with other materials, the V_max_ of TMB of Pt-BGP NCs was as high as 175.44 × 10^−8^ Ms^−1^, which illustrated that Pt-BGP NCs had the highest catalytic activity. This might be related to the ultra-small size of Pt NCs, and more Pt atoms were exposed on the surface of the material.

By comparison, it could be seen that Pt-BGP NCs accelerated the conversion of H_2_O_2_ to •OH radicals. Therefore, the catalytic mechanism of Pt-BGP NCs was due to the fact that Pt-BGP NCs accelerated the production of •OH radicals from H_2_O_2_, and then •OH radicals oxidized TMB to oxTMB. In the catalytic process, H_2_O_2_ might be adsorbed on the surface of Pt-BGP NCs. Then, the O-O bond of H_2_O_2_ could be decomposed into two •OH radicals. When TMB was present, •OH radicals easily reacted with TMB to produce blue oxTMB; otherwise, H_2_O_2_ would be decomposed into H_2_O and O_2_.

The detection range of Pt-BGP NCs for ascorbic acid was 2–100 μM, and the detection line was 0.191 μM. Compared with other components, the much higher ΔA of ascorbic acid indicated that the method was highly selective for ascorbic acid detection. After calculation, the mass of ascorbic acid in vitamin C (*Vc*) tablets was 98.97 mg, which was 98.97% of the true value. This result indicated that this method was reliable.

In addition, BGP and Pt-BGP NCs had no obvious cytotoxicity within 80 μg/mL. Yang et al. [31] used peppermint leaf extract to cap nanoparticles which showed different toxicity of Pt-BGP NCs. Cells maintained good cell activity when treated with peppermint extract within 200 μg/mL, while the capped nanoparticles showed good cell viability below 25 μg/mL. Table S1 shows that the biocompatibility of our prepared Pt-BGP NCs was much better than those of other materials. Hemolysis analysis indicated that there was extremely low effect between samples and red blood cells, and the cell membrane of red blood cells was basically intact. However, the hemolysis rate of ZnO nanoparticles (12 μg/mL) prepared by Zare et al. [32] reached 5%, which is slightly higher than that of Pt-BGP NCs. Thus, good biocompatibility of BGP and Pt-BGP NCs was due to the fact that BGP and Pt-BGP NCs had little effect on the cell membrane.

## 5. Conclusions

In this work, we successfully prepared Pt-BGP NCs using BGP and K_2_PtCl_4_. The size of Pt NCs inside of Pt-BGP NCs was 0.83 ± 0.22 nm. Pt-BGP NCs had excellent peroxidase-like activity. Pt-BGP NCs had a low ascorbic acid detection limit of 0.191 μM and good selectivity. The detection of real samples showed that it had excellent reliability. The biocompatibility experiment confirmed that Pt-BGP NCs had good biocompatibility. No noticeable cytotoxicity was measured when the concentration of Pt-BGP NCs was 80 μg/mL. In short, we developed a simple, low-cost, high-reliability, and biocompatible method of ascorbic acid detection, which may be used for the detection of ascorbic acid in food and drugs in the future.

## Figures and Tables

**Table 1:** Comparison of kinetic parameters of K_m_ and V_max_.

Catalysts	K_m_ (mM)		V_max_ (10^−8^ Ms^−1^)		References
	TMB	H_2_O_2_	TMB	H_2_O_2_	
Pt-BGP NCs	0.70	1.50	175.44	10.73	this work
Pt NPs	0.127	1.14	2	3.1	[20]
CeO_2_/C NWs	0.12	2.61	2.08	3.31	[19]
HPR	0.434	3.7	10.0	8.71	[21]
SiW_12_@Co_3_O_4_	0.023	167.8	5.3	25.1	[22]
Mo-CQDs NMs	0.38	0.05	19.5	22.8	[23]

**Table 2:** Comparison of linear range and detection limit of different peroxidase mimics.

Catalyst	Linear Range (μM)	LOD (μM)	References
Pt-BGP NCs	2–100	0.191	this work
PTCA-ZnFe_2_O_4_	1–10	0.834	[6]
AgFKZSiW_12_@PPy	1–80	2.7	[24]
SQE	50–425	10	[25]
Co_3_O_4_@β-CD NPs	10–60	1.09	[26]
CDs/Fe_3_O_4_ NFs	6.0–60	3.29	[27]

## Data Availability

The data presented in this study are available on request from the corresponding author.

## Supplementary Materials

The following are available online at https://www.mdpi.com/article/10.3390/biom11050647/s1, peroxidase-like activity assay of Pt-BGP NCs, biocompatibility of Pt-BGP NCs, Figure S1: protein content standard curve, Table S1: Comparison of the non-cytotoxic concentration of different mimic enzymes.
