# Supplementary material for: Biocompatible Platinum Nanoclusters Prepared Using Bitter Gourd Polysaccharide for Colorimetric Detection of Ascorbic Acid
Source: Biomolecules. 2021 Apr 28;11(5):647. doi: 10.3390/biom11050647 (PMC8146894; doi:10.3390/biom11050647)
Supplement: Supplementary file 1 [file biomolecules-11-00647-s001.zip › biomolecules-1195290-supplementary.pdf]

# Supporting information

# Biocompatible platinum nanoclusters prepared using bitter gourd polysaccharide for colorimetric detection of ascorbic acid

Kai Liu [1], Yu Zhao [1], Lu Zhang [1], Mengmeng He [1], Weifeng Lin [2], Haotian Sun [3], Zhiwei Liu [1], Jie Hu [1], Longgang Wang [1*]

*1 State Key Laboratory of Metastable Materials Science and Technology, Key Laboratory of Applied Chemistry, Hebei Key Laboratory of heavy metal deep-remediation in water and resource reuse, Yanshan University, Qinhuangdao, 066004, China.*

*2 Department of Materials and Interfaces, Weizmann Institute of Science, Rehovot 76100, Israel*

*3 Ocean NanoTech, LLC, San Diego, CA 92126, USA*

## 1 Peroxidase-like activity assay of Pt-BGP NCs

The peroxidase activity of Pt-BGP NCs was determined by detecting the amount of TMB oxidized to oxTMB. Three sets of experiments were designed to study the catalytic activity of Pt-BGP NCs: (i) TMB+Pt-BGP NCs; (ii) TMB+$H_2O_2$; (iii) TMB+Pt-BGP NCs+ $H_2O_2$. In the last set of experiments, 800 μL of TMB (0.6 mM), 200 μL of Pt-BGP NCs ($C_{Pt}$=0.66 mM) and 100 μL of $H_2O_2$ (0.3 M) were added to a 2ml PE tube and reacted at 30°C for 5 min. Then the absorbance was measured with a UV-Vis photometer. The experiment steps of the other groups were similar.

The effect of pH on the catalytic activity of Pt-BGP NCs was studied. 800 μL of TMB (0.6 mM) in different pH buffer solutions (pH=1-12), 200 μL of Pt-BGP NCs ($C_{Pt}$ = 0.66 mM) and 300 μL of buffer solution were mixed in a 2ml PE tube at 30°C for 5 min. 100 μL $H_2O_2$ (0.3 M) was mixed in the above solution at 30°C for 5 min, and finally the absorbance was measured at 652 nm by UV-Vis spectroscopy. The reaction process affected by temperature is similar to the above process. The pH value of the solution is fixed at pH=4, and the temperature is 20 to 80°C.

The catalytic kinetics of Pt-BGP NCs was Studied. Catalytic kinetics of TMB: 200 μL of Pt-BGP NCs ($C_{Pt}$ = 0.66 mM) and 200-1000 μL TMB (pH=4, 0.6 mM) were mixed at 30°C for 5 min, and then

100 μL $H_2O_2$ (0.3 M) was mixed in above solution. Finally, UV-Vis spectroscopy was used to record the absorbance of the experimental sample at 652 nm. The catalytic kinetics of $H_2O_2$ was measured by changing the concentration of $H_2O_2$. The other steps are similar to the catalytic kinetics of TMB. The parameters $K_m$ and $V_{max}$ were figure out by calculation of the Michaelis-Menten equation (1).

$$V = V_{max} \frac{[S]}{K_m + [S]} \quad (1)$$

## 2 Biocompatibility of Pt-BGP NCs

HeLa cells was evenly inoculated in a 96-well tissue culture plate (6000 cells/well) at $CO_2$ concentration of 5%, 37°C, and 200 μL of high glucose-sugar medium containing 10% fetal bovine serum (FBS) for 24 h. Then, the medium was changed to a high-glucose medium containing 200 μL of different BGP and Pt-BGP NCs concentrations in the same environment for 24 h. Then the medium was changed to 100 μL thiazole blue solution (C=0.5 mg/mL) for 4 h. The thiazole blue solution was substituted for 150 μL DMSO. Finally, the absorbance was read by Spectra Max M2 at 490 nm and calculate the cell viability.

## 3 Results and discussion

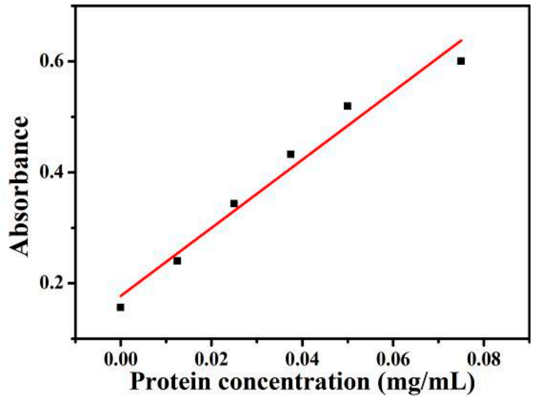


**Figure S1.** Protein content standard curve

**Table S1** Comparison of the non-cytotoxic concentration of different mimic enzymes

| Catalysts | Non-cytotoxic concentration (μg/mL) | Reference |
|---|---|---|
| Pt-BGP NCs | 80 | This work |
| Au-Pt/SiO2 NPs | 25 | (Wu et al., 2017) |
| Pt/P NPs | 25 | (Yang et al., 2017) |
| Au@Pt NDRs | 20 | (J. Liu et al., 2015) |

## References


1. Zare, M.; Namratha, K.; Thakur, M. S.; & Byrappa, K. Biocompatibility assessment and photocatalytic activity of bio-hydrothermal synthesis of ZnO nanoparticles by Thymus vulgaris leaf extract. *Materials Research Bulletin.* 2019,109, 49-59.
2. Zhao, L.; Wu, Z.; Liu, G.; Lu, H.; Gao, Y.; Liu, F.; Lu, G. High-activity Mo, S co-doped carbon quantum dot nanozyme-based cascade colorimetric biosensor for sensitive detection of cholesterol. *J Mater Chem B.* 2019, 7(44), 7042-7051.
3. Zhao, X.; Wu, K.; Lyu, H.; Zhang, X.; Liu, Z.; Fan G.; Liu, Q. Porphyrin functionalized $Co(OH)_2$/GO nanocomposites as an excellent peroxidase mimic for colorimetric biosensing. *Analyst.* 2019, 144(17), 5284-5291.
